# Neuritogenic and Neuroprotective Properties of Peptide Agonists of the Fibroblast Growth Factor Receptor

**DOI:** 10.3390/ijms11062291

**Published:** 2010-05-26

**Authors:** Shizhong Li, Elisabeth Bock, Vladimir Berezin

**Affiliations:** Protein Laboratory, Department of Neuroscience and Pharmacology, Panum Institute, University of Copenhagen, Copenhagen N, Denmark; E-Mails: li@sund.ku.dk (S.L.); bock@sund.ku.dk (E.B.)

**Keywords:** FGF, FGFR, NCAM, FGFR agonist, neuroprotective effect

## Abstract

Fibroblast growth factor receptors (FGFRs) interact with their cognate ligands, FGFs, and with a number of cell adhesion molecules (CAMs), such as the neural cell adhesion molecule (NCAM), mediating a wide range of events during the development and maintenance of the nervous system. Determination of protein structure, *in silico* modeling and biological studies have recently resulted in the identification of FGFR binding peptides derived from various FGFs and NCAM mimicking the effects of these molecules with regard to their neuritogenic and neuroprotective properties. This review focuses on recently developed functional peptide agonists of FGFR with possible therapeutic potential.

## 1. Introduction

### 1.1. FGF Receptors

Fibroblast growth factors (FGFs) exert their effects in target cells by signaling through cell-surface receptor tyrosine kinases. Five FGF receptors (FGFRs), FGFR1-5, have been identified. FGFRs contain three extracellular immunoglobulin (Ig)-like modules, an acidic region located between Ig1 and Ig2, a single transmembrane domain, and an intracellular split tyrosine kinase domain [1]. FGFR5 does not contain an active tyrosine kinase domain. Alternative mRNA splicing results in a number of FGFR splice variants. The third Ig module of FGFR1-3 is encoded by the invariant exon IIIa followed by one of two alternative exons, IIIb or IIIc [2]. Alternate usage of exons IIIb or IIIc affects the specificity of FGF binding to FGFRs. For example, FGF7 and FGF10 are known to bind FGFR2 IIIb but not FGFR2 IIIc [3,4].

Binding of FGFs to FGFRs induces FGFR dimerization, leading to receptor autophosphorylation [5]. Autophosphorylation of tyrosine residues in the intracellular domain of FGFR results in the docking of FGF receptor substrate 2α (FRS2α), phospholipase-Cγ (PLCγ), and Src homologous and collagenA (ShcA) and subsequent activation of various intracellular signaling pathways, including the mitogen-activated protein (MAP) kinase, calcium/calmodulin dependent protein kinase, and phosphoinositide-3 (PI3) kinase pathways [6,7].

### 1.2. Ligands of FGF Receptors

#### 1.2.1. FGFs

FGFs comprise a large family of 22 structurally and functionally related polypeptide growth factors. In vertebrates, the molecular mass of FGFs ranges from 17 to 34 kDa. FGFs possess a central core of 140 amino acids containing 12 antiparallel β-strands in which the sequence similarity between different members is 30–60% [8]. FGFs bind heparin and heparan sulfate proteoglycans with high affinity [9]. Several basic amino-acid residues in the β10-β11 strands of the growth factors are supposed to be involved in heparin binding [10]. This interaction is hypothesized to be important for FGF stabilization and FGFR activation [11,12].

Among the 22 FGF members, at least 10 are expressed in the brain. FGF1 is predominantly expressed in neurons and is involved in neuroprotection, learning, and memory. FGF2 is expressed in both neurons and astrocytes and is involved in neurogenesis, axonal growth, neuroprotection, and regeneration. FGF1 knockout mice are normal in appearance and behavior [13]. FGF2 knockout mice are viable but exhibit distinct defects in the organization of cortical neurons [14]. Our knowledge of the roles of other FGFs expressed in the central nervous system is currently limited [15]. FGF6 knockout mice are viable, and they display only mild disturbances in muscle regeneration [16]. FGF8 knockout is lethal and is characterized by mid-hindbrain boundary defects [17] and disturbed cerebellar development [18]. FGF8 is also required for the survival of nephrons [19]. FGF9 has been suggested to be a survival factor for medial thoracic and sacral motoneurons and retinal ganglion cells [20,21]. FGF17-deficient mice display disturbed cerebellar development [18].

#### 1.2.2. Cell Adhesion Molecules

In addition to the cognate ligands, the FGFs, FGFR interacts with a number of cell adhesion molecules (CAMs), including the neural cell adhesion molecule (NCAM), *N*-cadherin, L1, neurofascin, and neuroplastin [22–25]. NCAM, *N*-cadherin, and L1 all promote neuronal differentiation as reflected by neurite outgrowth following homophilic binding to the same CAM expressed on the responsive cell [26]. The possible involvement of FGFR in CAM-mediated induction of neuronal differentiation has been suggested by the observation that a specific tyrosine kinase inhibitor blocked the neurite outgrowth response to all three CAMs at an early stage in the signal transduction cascade. A so-called CAM homology domain in FGFR1 (*i.e.*, a stretch of 20 amino acids residing in the IgII module) has been suggested to be involved in CAM binding [27–30]. This sequence is highly conserved between all FGFRs and has sequence homology with *N*-cadherin, NCAM, and L1 [29]. This review focuses primarily on NCAM interactions with FGFR because many of the FGFR peptide agonists have been derived from this molecule.

NCAM plays multiple roles in nervous system development and maintenance [31–34]. NCAM also modulates neuronal plasticity, mediating learning and memory [35–37]. Studies in NCAM knockout mice show that NCAM is crucial for the formation of the olfactory bulb and the mossy fiber system in the hippocampus [38,39]. Polysialic acid (PSA) is a linear homopolymer containing α2, eight sialic acid residues, and NCAM has been shown to be the main PSA carrier in vertebrates. The polysialylated form of NCAM (PSA-NCAM) plays an important role in neural plasticity, including axonal growth, cell migration, synaptic plasticity, and embryonic and adult neurogenesis [40]. Slices prepared from either NCAM knockout mice or mice treated with a PSA-removing enzyme, Endo N, display defects in long-term potentiation (LTP) in the CA1 region of the hippocampus [41].

NCAM is a member of the immunoglobulin (Ig) superfamily (IgSF) and was first described as a synaptosomal membrane protein, termed D2 [42]. NCAM is widely expressed in the developing nervous system and is also found in muscles, heart, kidneys, and gonads. The extracellular part of NCAM is composed of five Ig modules (Ig1–Ig5) and two fibronectin type III (FN3) modules, FN3(1) and FN3(2). In the nervous system three major isoforms are expressed, NCAM-120, NCAM-140, and NCAM-180 (the numbers corresponding to their relative molecular weights), resulting from alternative splicing of a single gene. NCAM-140 and NCAM-180 are transmembrane isoforms that differ in the size of their cytoplasmic domains, whereas NCAM-120 is attached to the membrane via a glycosylphosphatidylinositol (GPI) anchor lacking the cytoplasmic domains [43]. By means of nuclear magnetic resonance (NMR) spectroscopy and X-ray crystallography, the structures of the NCAM Ig1–3, FN3(1), and FN3(2) modules have been determined [44–46].

Direct binding of the two NCAM FN3 modules to the second and third FGFR1 Ig modules has been demonstrated by surface plasmon resonance (SPR) analysis [45]. The NCAM FN3 modules can mimic the neuritogenic activity of the intact molecule [47,48], presumably via interactions with FGFR. Moreover, the second NCAM FN3 module has been shown to induce FGFR1 phosphorylation and promote neurite outgrowth in an FGFR activation-dependent fashion. An NCAM binding site for FGFR1 has been mapped by NMR titration analysis to a region in the second FN3 module [45]. This region also has sequence and structural homology with a part of FGF2.

## 2. Peptide Agonists of FGFR Derived from FGFs

During the past few years, several peptide agonists of FGFR have been identified based on analyses of the interaction interfaces between FGFs and FGFRs as they appear in the crystal structure of the FGF-FGFR complexes. Other FGFR binding peptides have been identified based on analyses of sequence and structure homologies between FGFs and CAMs.

### 2.1. Canofins: Peptide Agonists of FGFR Derived from the Canonical FGFR Binding Sites

**Figure 1.**
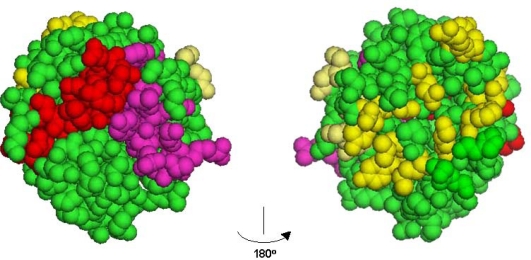
A space-filling model of FGF2 (PDB:1CVS). Two 180° rotation projections are shown. The sequence motifs of hexafin and dekafin are mapped in red and magenta, respectively. The residues constituting the primary and secondary FGFR binding sites [49] are shown in yellow. The figure was made using PyMOL Molecular Viewer (DeLano Scientific LLC, San Francisco, CA, USA).

According to the crystal structure of the FGF2-FGFR1c complexes, FGF2 forms contacts with Ig2, Ig3, and the interconnecting linker of FGFR1c. The FGF2 residues involved in interactions with Ig2 of the receptor have been shown to be located in the β1, β2, β3, and β12 strands and in the β1–β2 and β8–β9 loops. The FGF2 residues located in the *N*-terminus, the β4, β5, and β8 strands, and the β7–β8 loop are involved in interactions with Ig3. The FGF2 residues interacting with the Ig2–Ig3 linker interface are located in the β8–β9 loop and β9 strand [49]. All binding interfaces with FGFR1c are generally located on one side of the FGF2 globular structure (Figure 1, yellow).

Three peptides, termed canofin1, canofin2, and canofin3, have been derived from the canonical binding sites of FGF2 encompassing the β1, β2, and β11–β12 loop-strand regions of the growth factor, respectively. The canofin peptides have been demonstrated to specifically bind to FGFR1 and induce FGFR1 phosphorylation. Moreover, phosphorylation induced by the cognate ligand, FGF2, is inhibited by all three canofins, suggesting that these peptides are partial agonists of FGFR [50]. Canofins strongly induce neurite outgrowth from primary cerebellar granule neurons in an FGFR activation-dependent manner. They have also been shown to be anti-apoptotic, promoting the survival of cerebellar granule neurons (Table 1). Canofins are regarded as valuable pharmacological tools for the study of the functional roles of specific interactions of FGF2 with FGFR1c [50].

### 2.2. Hexafins: Peptide Agonists of FGFR Derived from the β6–β7 Loop Regions of Various FGFs

The hexafin peptides have been identified based on analysis of the structural homology between a sequence motif in the second NCAM FN3 module involved in NCAM-FGFR interactions, and the β6–β7 loop region of FGF2 [45]. All 22 FGFs share homologous sequence motifs located in the β6–β7 region [51], and this motif has subsequently been termed the hexafin motif. In the space-filling model of FGF2 in Figure 1 the hexafin motif is marked in red in the β6–β7 region. This motif is located on the FGF2 side opposite to the FGF2-FGFR1c binding interface observed in the crystal structure [49]. Hexafins derived from FGF1, 2, 3, 8, 9, 10 and 17 have been characterized and found to bind to FGFR1IIIc and FGFR2IIIb, respectively, with kD values ranging from 10^−7^ to 10^−8^ M. Moreover, these peptides have been shown to activate FGFR1-IIIc. Hexafins derived from FGF2, 3, 8, 10, and 17, but not from FGF1 and 9, promote neurite extension, and hexafins derived from FGF1, 3, 9, 10, and 17, but not from FGF2 and 8, promote neuronal survival [51]. Furthermore, *in vivo* studies show that treatment with hexafin1 and hexafin2 results in prolonged retention of social memory in adult rats, and rats treated with hexafin2 exhibit decreased anxiety-like behavior in the elevated plus maze. Hexafin2 has also been shown to be able to alleviate deficits in activity related to social behavior in the R6/2 mouse model of Huntington’s disease (Table 1) [52].

### 2.3. Dekafins: Peptide Agonists of FGFR Derived from the β10–β11 Loop Regions of Various FGFs

The dekafin peptides were identified based on sequence homology between a sequence in the first NCAM FN3 module and a sequence motif located in the β10–β11 loop regions of all FGFs but FGF19, 21, 22 and 23 (in human). This motif has subsequently been termed the dekafin motif. Dekafins derived from FGF1, 2, 3, 5, 6, 8, 9, 10, and 17 have been characterized and found to bind to FGFR1IIIc and FGFR2IIIb, respectively, with kD values ranging from 10^−7^ to 10^−8^ M [ 52]. Figure 1 shows the model of FGF2 with the dekafin motif marked in magenta in the β10–β11 region. Similar to the hexafin motif, the dekafin sequence is located on the FGF2 side opposite to the FGF2-FGFR1 binding interface observed in the crystal structure [49]. A number of basic residues have been shown to be necessary for dekafin1 interactions with FGFR1c. These residues are known to be involved in heparin binding, and heparin analogs have been shown to inhibit dekafin1 binding to the receptor [53]. Dekafin1, 2, 3, 5, 6, 8, 9, 10, and 17 all induce FGFR1c phosphorylation in TREX cells and neurite outgrowth in primary cerebellar granule neurons, although with different potencies. Dekafins are partial agonists of FGFR as reflected by their inhibition of receptor activation induced by the cognate ligand, FGF1. The neuritogenic effect of dekafin1, 2, and 10 has been shown to be sensitive to treatment with a pharmacological inhibitor of FGFR, and dekafin6, 8, 9, and 17 have been demonstrated to be neuroprotective *in vitro* (Table 1).

## 3. Peptide Agonists of FGFR Derived from NCAM

**Figure 2.**
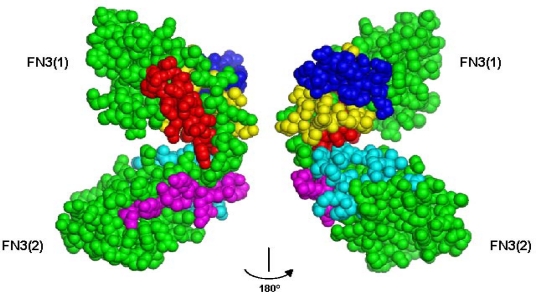
A space-filling model of the two NCAM FN3 modules. Two 180° rotation projections are shown. The sequence motifs of EnkaminA, EncaminC, EncaminE, FGL, and BCL are mapped in red, blue, yellow, magenta, and cyan, respectively. The figure was made using PyMOL Molecular Viewer (DeLano Scientific LLC, San Francisco, CA, USA).

Interactions between NCAM with FGFR occur through binding of the two most membrane-proximal NCAM modules, FN3 modules 1 and 2. Both NCAM FN3(1) and FN3(2) have been shown by surface plasmon resonance analysis to be involved in binding to an Ig2–Ig3 construct of FGFR1 and FGFR2, both splice variant IIIc [54]. A number of synthetic peptides have been synthesized based on sequence motifs in the FN3 modules of NCAM and been found to interact with FGFR. The peptide positions are shown in Figure 2.

### 3.1. Peptide Agonists of FGFR Derived from the First NCAM FN3 Module

All FN3 modules have a similar topology. Their tertiary structure is composed of two opposing β-sheets, each containing three to four β-strands and the interconnecting loops. To identify peptide mimetics with the potential to interact with FGFR, a strand-loop-strand strategy has been used [55]. Following this strategy, six peptides sequentially encompassing the AB-, BC-, CD-, DE-, EF-, and FG-strand-loop-strand regions have been synthesized and tested for their ability to bind FGFR1 and induce FGFR1 phosphorylation. The active peptides derived from the AB-, CD-, and EF-loop regions were termed EncaminA, C, and E, respectively [55]. The active peptide derived from the FG-loop region is termed dekaCAM [53].

EncaminA, C, and E are all located in the *C*-terminal part of the folded NCAM FN3(1) module. They bind to and activate FGFR1. The kD values range from 10^−8^ to 10^−7^ M. EncaminA and E, but not C, activate Akt/PKB, and EncaminE, but not A and C, activates Erk in primary hippocampal neurons. EncaminC and E, but not A, have been shown to induce neurite outgrowth and promote the survival of cerebellar granule neurons. They also enhance presynaptic function *in vitro* as reflected by the increased rate of transmitter relaese (Table 1) [55]. The Encamin sequence partially overlaps the FRM motif, which has been previously shown to be able to stimulate neuronal differentiation and neuroprotection [56].

### 3.2. Peptide Agonists of FGFR Derived from the Second NCAM FN3 Module

The structure of the second NCAM FN3 module has been solved using NMR spectroscopy (as a single module [45]) and X-ray crystallography (together with FN3[1]) [46]. One of the FN3(2) strand-loop-strand regions, the FG loop motif (FGL), has been mapped by NMR titration analysis as an NCAM binding site for FGFR [45].

The FGL peptide has since been the subject of extensive *in vitro* and *in vivo* studies, which have established this FGFR agonist as a pharmacological mimetic of various NCAM-related functional modalities (Table 1). FGL has been demonstrated *in vitro* to bind to and activate FGFR1 and downstream signaling molecules and cascades, such as FRS2α, ShcA, PLCγ, Akt/PKB, and Erk1/2 [57,58], induce differentiation of primary neurons [45,58], promote neuronal survival [58], promote synapse formation, enhance presynaptic function [36], and attenuate the impact of inflammation [59]. FGL is able to enhance spatial and social memory in normal animals *in vivo*, promote postnatal sensorimotor development [60], protect hippocampal neurons against ischemic insult [61], and ameliorate cognitive deficits and reduce neuropathological signs in a model of β-amyloid peptide-induced neurotoxicity, possibly by inhibition of GSK3β [62]. Moreover, FGL has been shown to modulate the transcriptional response to traumatic brain injury [63], attenuate age-related changes in LTP and inflammatory signs [64], and prevent stress-induced dementia, possibly by inducing substantial changes in the fine structure of synapses and dendritic spines in the hippocampus [65,66]. FGL has also been shown to reverse a depression-like phenotype in NCAM-deficient mice and ameliorate working memory deficits in a rat model of schizophrenia [67].

Importantly, the FGL peptide has been shown to rapidly reach the blood and cerebrospinal fluid after systemic administration [60]. FGL has shown no adverse effects in rats, dogs, monkeys (preclinical studies), and healthy human male volunteers [68]. Thus, the pharmacological properties of FGL indicate that this peptide can target neurodegenerative and cognitive disorders.

In addition to FGL, peptides derived from other strand-loop-strand regions in the NCAM FN3(2) module, namely ABL, BCL, CDL, DEL, and EFL, have been synthesized and tested for their capability to bind to FGFR. The BCL peptide was found to be the only peptide that binds to FGFR1 [69]. Similar to FGL, the BCL peptide has been demonstrated to activate the receptor and induce a neuritogenic response. Unlike FGL, however, BCL does not promote neuronal survival [69].

## 4. Concluding Remarks

Identification of binding sites by structural studies or by *in silico* molecular modeling allows the development of functional agonists or antagonists of cell surface receptors. Thus, peptides derived from FGFR binding site of various FGFs and NCAM have been shown to act as agonists of the receptor, mimicking the functions of these molecules. The effects of the majority of the peptide mimetics presented in Table 1 have been studied using peptides synthesized as dimers or tetramers. A dimer or tetramer promote receptor dimerization and thereby activation. However, mimetic peptides are usually much less potent than the growth factors from which the peptides are derived.

**Figure 3.**
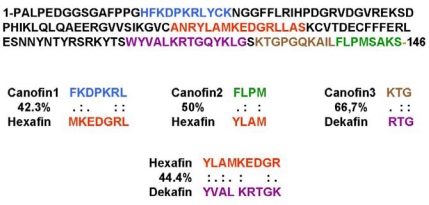
Sequence of human FGF2 with the canofin1, canofin2, canofin3, hexafin, and dekafin motifs marked in blue, green, brown, red, and magenta, respectively. Sequence identities are indicated as a percentage of amino-acid overlap.

Not very surprisingly, a sequence motif engaged in protein-protein interactions observed in a crystal structure is capable of mimicking the functional activity of the protein when prepared as a synthetic peptide. Examples of such peptides are canofins [49]. Unexpectedly, however, *in silico* molecular modeling has also identified active motifs in FGFs that are located on the side of the growth factors that is opposite to the side shown by X-ray crystallography to be involved in receptor binding. Examples of such motifs are hexafins and dekafins (Figure 1), whose sequences actually are homologous to the canonical binding sites, such as the hexafin and dekafin motifs, see Figure 3. One explanation may be that the hexafin and dekafin motifs are remnants of the evolutionary process due to gene duplication. However, the hexafin and dekafin peptides show the same receptor binding and activation activity as the canofin peptides [51,53]. The non-canonical binding sites in the FGFs may also play a role in FGF interactions with FGFR, probably depending on the specific cellular context. An alternative explanation may be that the non-canonical binding sites in the FGFs serve to pre-concentrate the growth factor in close proximity to the FGFRs until it is positioned most favorably for high affinity interactions involving the canonical binding sites. Further studies employing FGF2 mutated on various FGFR binding sites will clarify this issue.

NCAM-FGFR interaction studies reveal multiple binding sites localized on both sides of the interface in the vicinity of the contact between the two NCAM FN3 modules (Figure 2). This implies that one NCAM molecule might concurrently interact with two FGFR molecules, thus promoting receptor dimerization and activation, when NCAM clustering is induced by homophilic NCAM adhesion.

Regardless of the precise roles of multiple FGFR binding motifs in FGFs and NCAM, identification of functional FGFR peptide agonists opens new possibilities for the development of pharmacological tools to study the molecular mechanisms underlying FGFR activation and signaling. Additionally, some of the agonists listed in Table 1 have apparent therapeutic potential.

## Figures and Tables

**Table 1.** Peptide agonists of FGFR derived from FGFs and NCAM.

**Peptide**	**Sequence motif localization**	***In vitro*effects**	***In vivo*effects**	**Refs.**
Canofin1, 2, and 3	Interaction sites between FGF2 and FGFR1	Induction of neurite outgrowth by binding to and activating FGFR. Protection against apoptosis.	N.D.	[50]
Hexafin1 and 9	β6–β7 regions of FGFs	Binding to and activation of FGFR. Protection against apoptosis.	Prolonged retention of social memory in adult rats.	[51,52]
Hexafin2	β6–β7 regions of FGFs	Induction of neurite outgrowth by binding to and activating FGFR.	Prolonged retention of social memory and decreased anxiety-like behavior in adult rats. Alleviation of deficits in activity related to social behavior, including sociability and social novelty, in R6/2 mouse model of Huntington’s disease.	[51,52]
Hexafin3, 10, and 17	β6–β7 regions of FGFs	Induction of neurite outgrowth by binding to and activating FGFR. Protection against apoptosis.	N.D.	[51]
Hexafin8	β6–β7 regions of FGFs	Induction of neurite outgrowth by binding to and activating FGFR.	N.D.	[51]
Dekafin1, 2, 3, 5, and 10	β10–β11 regions of FGFs	Induction of neurite outgrowth by binding to and activating FGFR.	N.D.	[53]
Dekafin6, 8, 9, and 17	β10–β11 regions of FGFs	Induction of neurite outgrowth by binding to and activating FGFR. Protection against apoptosis.	N.D.	[53]
DekaCAM	β10–β11 regions of FGFs	Induction of neurite outgrowth by binding to and activating FGFR.	N.D.	[53]
FRM	NCAM/FN3(1)	Stimulation of differentiation and neuroprotection.	N.D.	[56]
EncaminA	NCAM/FN3(1)	Induction of neurite outgrowth by binding to and activating FGFR and Akt/PKB.	N.D.	[55]
EncaminC	NCAM/FN3(1)	Induction of neurite outgrowth by binding to and activating FGFR and Erk. Protection against apoptosis. Enhancement of presynaptic function.	N.D.	[55]
EncaminE	NCAM/FN3(1)	Induction of neurite outgrowth by binding to and activating FGFR and Akt/PKB. Protection against apoptosis. Enhancement of presynaptic function.	N.D.	[55]
FGL	NCAM/FN3(2)	Induction of neurite outgrowth by binding to and activating FGFR followed by activation of ERK1/2 and Akt/PKB. Activation of FRS2α, ShcA, and PLCγ. Protection against apoptosis. Promotion of synapse formation. Enhancement of presynaptic function. Attenuation of inflammatory impact.	Enhancement of spatial and social memory. Promotion of postnatal sensorimotor development. Protection of hippocampal neurons against ischemic insult. Reduction of neuropathological signs and cognitive impairment in Alzheimer’s disease model. Modulation of the transcriptional response to traumatic brain injury. Attenuation of age-related changes in long-term potentiation and inflammatory signs and prevention of stress-induced dementia. Induction of large changes in the fine structure of dendritic spines in the hippocampus of aged rats related to improved cognitive function. Reversal of depression-like phenotype in NCAM-deficient animals. Amelioration of working memory deficits in rats after neonatal phencyclidine treatment.	[36,45, 54,58–68]
BCL	NCAM/FN3(2)	Binding to and activating of FGFR1. Stimulation of neurite outgrowth.	N.D.	[69]

## References

[1] Powers CJ McLeskey SW Wellstein A Fibroblast growth factors, and their receptors and signaling Endocr. Relat. Cancer 2000 7 165 197 11021964 10.1677/erc.0.0070165

[2] Goldstrohm AC Greenleaf AL Garcia-Blanco MA Co-transcriptional splicing of pre-messenger RNAs: Considerations for the mechanism of alternative splicing Gene 2001 277 31 47 11602343 10.1016/s0378-1119(01)00695-3

[3] Orniz DM FGFs, heparan sulfate and FGFRs: Complex interactions essential for development Bioessays 2000 22 108 112 10655030 10.1002/(SICI)1521-1878(200002)22:2<108::AID-BIES2>3.0.CO;2-M

[4] Yeh BK Igarashi M Eliseenkova AV Plotnikov AN Sher I Ron D Aaronson SA Mohammadi M Structural basis by which alternative splicing confers specificity in fibroblast growth factor receptors Proc. Natl. Acad. Sci. USA 2003 100 2266 2271 12591959 10.1073/pnas.0436500100 PMC151329

[5] Mohammadi M Olsen SK Ibrahimi OA Structural basis for fibroblast growth factor receptor activation Cytokine Growth Factor Rev 2005 16 107 137 15863029 10.1016/j.cytogfr.2005.01.008

[6] Eswarakumar VP Lax I Schlessinger J Cellular signaling by fibroblast growth factor receptors Cytokine Growth Factor Rev 2005 16 139 149 15863030 10.1016/j.cytogfr.2005.01.001

[7] Dailey L Ambrosetti D Mansukhani A Basilico C Mechanisms underlying differential responses to FGF signaling Cytokine Growth Factor Rev 2005 16 233 247 15863038 10.1016/j.cytogfr.2005.01.007

[8] Zhang J Cousens LS Barr PJ Sprang SR Three-dimensional structure of human basic fibroblast growth factor, a structural homolog of interleukin 1β Proc. Natl. Acad. Sci. USA 1991 88 3446 3450 1849658 10.1073/pnas.88.8.3446 PMC51464

[9] Burgess WH Maciag T The heparin-binding (fibroblast) growth factor family of protein Ann. Rev. Biochem 1989 58 575 606 2549857 10.1146/annurev.bi.58.070189.003043

[10] Zhu X Komiya H Chirino A Faham S Fox GM Arakawa T Hsu BT Rees DC Three-dimensional structures of acidic and basic fibroblast growth factors Science 1991 251 90 93 1702556 10.1126/science.1702556

[11] Ornitz DM Itoh N Fibroblast growth factors Genome Biol 2001 2 3005:1 3005:12 10.1186/gb-2001-2-3-reviews3005 PMC138918 11276432

[12] Ornitz DM Xu J Colvin JS McEwen DG MacArthur CA Coulier F Gao G Goldfarb M Receptor specificity of the fibroblast growth factor family J. Biol. Chem 1996 271 15292 15297 8663044 10.1074/jbc.271.25.15292

[13] Miller DL Ortega S Bashayan O Basch R Basilico C Compensation by fibroblast growth factor 1 (FGF1) does not account for the mild phenotypic defects observed in FGF2 null mice Mol. Cell Biol 2000 20 2260 2268 10688672 10.1128/mcb.20.6.2260-2268.2000 PMC110842

[14] Raballo R Rhee J Lyn-Cook R Leckman JF Schwartz ML Vaccarino FM Basic fibroblast growth factor (Fgf2) is necessary for cell proliferation and neurogenesis in the developing cerebral cortex J. Neurosci 2000 20 5012 5023 10864959 10.1523/JNEUROSCI.20-13-05012.2000 PMC6772267

[15] Reuss B von Bohlen und Halbach O Fibroblast growth factors and their receptors in the central nervous system Cell Tissue Res 2003 313 139 157 12845521 10.1007/s00441-003-0756-7

[16] Fiore F Sebille A Birnbaum D Skeletal muscle regeneration is not impaired in *Fgf6* -/- mutant mice Biochem. Biophys. Res. Commun 2000 272 138 143 10872817 10.1006/bbrc.2000.2703

[17] Reifers F Bohli H Walsh EC Crossley PH Stainier DY Brand M Fgf8 is mutated in zebrafish acerebellar (*ace*) mutants and is required for maintenance of midbrain-hindbrain boundary development and somitogenesis Development 1998 125 2381 2395 9609821 10.1242/dev.125.13.2381

[18] Xu J Liu Z Ortitz DM Temporal and spatial gradients of Fgf8 and Fgf17 regulate proliferation and differentiation of midline cerebellar structures Development 2000 127 1833 1843 10751172 10.1242/dev.127.9.1833

[19] Grieshammer U Cebrian C Ilagan R Meyers E Herzlinger D Martin GR FGF8 is required for cell survival at distinct stages of nephrogenesis and for regulation of gene expression in nascent nephrons Development 2005 132 3847 3857 16049112 10.1242/dev.01944

[20] Garces A Nishimune H Philippe JM Pettmann B deLapeyriere O FGF9: A motoneuron survival factor expressed by medial thoratic and sacral motoneurons J. Neurosci. Res 2000 60 1 9 10723063 10.1002/(SICI)1097-4547(20000401)60:1<1::AID-JNR1>3.0.CO;2-P

[21] Kinkl N Ruiz J Vecino E Frasson M Sahel J Hicks D Possible involvement of a fibroblast growth factor 9 (FGF9)-FGF receptor-3-mediated pathway in adult pig retinal ganglion cell survival *in vitro* Mol. Cell Neurosci 2003 23 39 53 12799136 10.1016/s1044-7431(03)00070-8

[22] Saffell L Williams EJ Mason IJ Walsh FS Doherty P Expression of a dominant negative FGF receptor inhibits axonal growth and FGF receptor phosphorylation stimulated by CAMs Neuron 1997 18 231 242 9052794 10.1016/s0896-6273(00)80264-0

[23] Kulahin N Li S Hinsby A Kiselyov V Berezin V Bock E Fibronectin type III (FN3) modules of the neuronal cell adhesion molecule L1 interact directly with the fibroblast growth factor (FGF) receptor Mol. Cell Neurosci 2008 37 528 536 18222703 10.1016/j.mcn.2007.12.001

[24] Kirschbaum K Kriebel M Kranz EU Pötz O Volkmer H Analysis of non-canonical fibroblast growth factor receptor 1 (FGFR1) interaction reveals regulatory and activating domains of neurofascin J. Biol. Chem 2009 42 28533 28542 19666467 10.1074/jbc.M109.004440 PMC2781396

[25] Owczarek S Kiryushko D Larsen MH Kastrup JS Gajhede M Sandi C Berezin V Bock E Soroka V Neuroplastin-55 binds to and signals through the fibroblast growth factor receptor FASEB J 2010 24 1139 1150 19952283 10.1096/fj.09-140509

[26] Doherty P Walsh FS Signal transduction events underlying neurite outgrowth stimulated by cell adhesion molecules Curr. Opin. Neurobiol 1994 4 49 55 8173325 10.1016/0959-4388(94)90031-0

[27] Williams EJ Walsh FS Doherty P Tyrosine kinase inhibitors can differentially inhibit integrin-dependent and CAM stimulated neurite outgrowth J. Cell Biol1994a 124 1029 1037 8132706 10.1083/jcb.124.6.1029 PMC2119981

[28] Williams EJ Furness J Walsh FS Doherty P Characterisation of the second messenger pathway underlying neurite outgrowth stimulated by FGF Development1994b 120 1685 1693 8050374 10.1242/dev.120.6.1685

[29] Williams EJ Furness J Walsh FS Doherty P Activation of the FGF receptor underlies neurite outgrowth stimulated by L1, N-CAM, and N-cadherin Neuron1994c 13 583 594 7917292 10.1016/0896-6273(94)90027-2

[30] Saffell L Walsh FS Doherty P Expression of NCAM containing VASE in neurons can account for a developmental loss in their neurite outgrowth response to NCAM in a cellular substratum J. Cell Biol 1994 125 427 436 8163558 10.1083/jcb.125.2.427 PMC2120034

[31] Rønn LC Hartz BP Bock E The neural cell adhesion molecule (NCAM) in development and plasticity of the nervous system Exp. Gerontol 1998 33 853 864 9951628 10.1016/s0531-5565(98)00040-0

[32] Rønn LC Doherty P Holm A Berezin V Bock E Neurite outgrowth induced by a synthetic peptide ligand of neural cell adhesion molecule requires fibroblast growth factor receptor activation J. Neurochem 2000 75 665 671 10899941 10.1046/j.1471-4159.2000.0750665.x

[33] Berezin V Bock E Poulsen F The neural cell adhesion molecule NCAM Curr. Opin. Drug. Disc. Dev 2000 3 605 609 19649888

[34] Hinsby AM Berezin V Bock E Molecular mechanisms of NCAM function Front. Biosci 2004 9 2227 2244 15353284 10.2741/1393

[35] Cremer H Chazal G Carleton A Goridis C Vincent JD Lledo PM Long-term but not short-term plasticity at mossy fiber synapses is impaired in neural cell adhesion molecule-deficient mice Proc. Natl Acad. Sci. USA 1998 95 13242 13247 9789073 10.1073/pnas.95.22.13242 PMC23769

[36] Cambon K Hansen SM Venero C Herrero AI Skibo G Berezin V Bock E Sandi C A synthetic neural cell adhesion molecule mimetic peptide promotes synaptogenesis, enhances presynaptic function, and facilitates memory consolidation J. Neurosci 2004 24 4197 4204 15115815 10.1523/JNEUROSCI.0436-04.2004 PMC6729275

[37] Sandi C Stress, cognitive impairment and cell adhesion molecules Nat. Rev. Neurosci 2004 5 917 930 15550947 10.1038/nrn1555

[38] Cremer H Chazal G Goridis C Represa A NCAM is essential for axonal growth and fasciculation in the hippocampus Mol. Cell Neurosci 1997 8 323 335 9073395 10.1006/mcne.1996.0588

[39] Muller D Wang C Skibo G Toni N Cremer H Calaora V Rougon G Kiss JZ PSA-NCAM is required for activity-induced synaptic plasticity Neuron 1996 17 413 422 8816705 10.1016/s0896-6273(00)80174-9

[40] Rutishauser U Polysialic acid in the plasticity of the developing and adult vertebrate nervous system Nat. Rev. Neurosci 2008 9 26 35 18059411 10.1038/nrn2285

[41] Muller D Djebbara-Hannas Z Jourdain P Vutskits L Durbec P Rougon G Kiss JZ Brain-derived neurotrophic factor restores long-term potentiation in polysialic acid-neural cell adhesion molecule-deficient hippocampus Proc. Natl. Acad. Sci. USA 2000 97 4315 4320 10760298 10.1073/pnas.070022697 PMC18239

[42] Jørgensen OS Bock E Brain specific synaptosomal membrane proteins demonstrated by crossed immunoelectrophoresis J. Neurochem 1974 23 879 880 4430927 10.1111/j.1471-4159.1974.tb04419.x

[43] Walmod PS Kolkova K Berezin V Bock E Zippers make signals: NCAM-mediated molecular interactions and signal transduction Neurochem. Res 2004 29 2015 2035 15662836 10.1007/s11064-004-6875-z

[44] Thomsen NK Soroka V Jensen PH Berezin V Kiselyov VV Bock E Poulsen FM The three-dimensional structure of the first domain of neural cell adhesion molecule Nat. Struct. Biol 1996 3 581 585 8673600 10.1038/nsb0796-581

[45] Kiselyov VV Skladchikova G Hinsby AM Jensen PH Kulahin N Soroka V Pedersen N Tsetlin V Poulsen FM Berezin V Bock E Structural basis for a direct interaction between FGFR1 and NCAM and evidence for a regulatory role of ATP Structure 2003 11 691 701 12791257 10.1016/s0969-2126(03)00096-0

[46] Carafoli F Saffell JL Hohenester E Structure of the tandem fibronectin type 3 domains of neural cell adhesion molecule J. Mol. Biol 2008 2 524 534 18261743 10.1016/j.jmb.2008.01.030 PMC2267215

[47] Frei T von Bohlen und Halbach F Wille W Schachner M Different extracellular domains of the neural cell adhesion molecule (N-CAM) are involved in different functions J. Cell Biol 1992 118 177 194 1618903 10.1083/jcb.118.1.177 PMC2289517

[48] Kasper C Rasmussen H Kastrup JS Ikemizu S Jones EY Berezin V Bock E Larsen IK Structural basis of cell-cell adhesion by NCAM Nat. Struct. Biol 2000 7 389 393 10802736 10.1038/75165

[49] Plotnikov AN Schlessinger J Hubbard SR Mohammadi M Structural basis for FGF receptor dimerization and activation Cell 1999 98 641 650 10490103 10.1016/s0092-8674(00)80051-3

[50] Manfe V Kochoyan A Bock E Berezin V Peptides derived from specific interaction sites of the fibroblast growth factor (FGF) 2-FGF receptor complexes induce receptor activation and signalling J Neurochem 2010 in press 10.1111/j.1471-4159.2010.06718.x 20374425

[51] Li S Christensen C Køhler LB Kiselyov VV Berezin V Bock E Agonists of fibroblast growth factor receptor induce neurite outgrowth and survival of cerebellar granule neurons Dev. Neurobiol 2009 69 837 854 19634127 10.1002/dneu.20740

[52] Rudenko O Tkach V Berezin V Bock E Effects of FGF receptor peptide agonists on animal behavior under normal and pathological conditions Neurosci Res 2010 in press 10.1016/j.neures.2010.05.002 20562017

[53] Li S Christensen C Kiselyov VV Køhler LB Bock E Berezin V Fibroblast growth factor-derived peptides: Functional agonists of the fibroblast growth factor receptor J. Neurochem 2008 104 667 682 18199118 10.1111/j.1471-4159.2007.05070.x

[54] Christensen C Lauridsen JB Berezin V Bock E Kiselyov VV The neural cell adhesion molecule binds to fibroblast growth factor receptor 2 FEBS Lett 2006 580 3386 3390 16709412 10.1016/j.febslet.2006.05.008

[55] Hansen SM Køhler LB Li S Kiselyov V Christensen C Owczarek S Bock E Berezin V NCAM-derived peptides function as agonist for the firoblast growth factor receptor J. Neurochem 2008 106 2030 2041 18624916 10.1111/j.1471-4159.2008.05544.x

[56] Anderson AA Kendal CE Garcia-Maya M Kenny AV Morris-Triggs SA Wu T Reynolds R Hohenester E Saffell JL A peptide from the first fibronectin domain of NCAM acts as an inverse agonist and stimulates FGF receptor activation, neurite outgrowth and survival J. Neurochem 2005 95 570 583 16135080 10.1111/j.1471-4159.2005.03417.x

[57] Chen Y Li S Berezin V Bock E The fibroblast growth factor receptor (FGFR) agonist FGF1 and the neural cell adhesion molecule-derived peptide FGL activate FGFR substrate 2α differently J. Neurosci. Res 2010 88 1882 1889 20175207 10.1002/jnr.22374

[58] Neiiendam JL Kohler LB Christensen C Li S Pedersen MV Ditlevsen DK Kornum MK Kiselyov VV Berezin V Bock E An NCAM-derived FGF-receptor agonist, the FGL-peptide, induces neurite outgrowth and neuronal survival in primary rat neurons J. Neurochem 2004 91 920 935 15525346 10.1111/j.1471-4159.2004.02779.x

[59] Downer EJ Cowley TR Cox F Maher FO Berezin V Bock E Lynch MA A synthetic NCAM-derived mimetic peptide, FGL, exerts anti-inflammatory properties via IGF-1 and interferon-γ modulation J. Neurochem 2009 109 1516 1525 19457161 10.1111/j.1471-4159.2009.06076.x

[60] Secher T Novitskaia V Berezin V Bock E Glenthoj B Klementiev B A neural cell adhesion molecule-derived fibroblast growth factor receptor agonist, the FGL-peptide, promotes early postnatal sensorimotor development and enhances social memory retention Neuroscience 2006 141 1289 1299 16784819 10.1016/j.neuroscience.2006.04.059

[61] Skibo GG Lushnikova IV Voronin KY Dmitrieva O Novikova T Klementiev B Vaudano E Berezin VA Bock E A synthetic NCAM-derived peptide, FGL, protects hippocampal neurons from ischemic insult both *in vitro* and *in vivo* Eur. J. Neurosci 2005 22 1589 1596 16197499 10.1111/j.1460-9568.2005.04345.x

[62] Klementiev B Novikova T Novitskaya V Walmod PS Dmytriyeva O Pakkenberg B Berezin V Bock E A neural cell adhesion molecule-derived peptide reduces neuropathological signs and cognitive impairment induced by Aβ_25–35_ Neuroscience 2007 145 209 224 17223274 10.1016/j.neuroscience.2006.11.060

[63] Pedersen MV Helweg-Larsen RB Nielsen FC Berezin V Bock E Penkowa M The synthetic NCAM-derived peptide, FGL, modulates the transcriptional response to traumatic brain injury Neurosci. Lett 2008 437 148 153 18436381 10.1016/j.neulet.2008.03.070

[64] Downer EJ Cowley TR Lyons A Mills KH Berezin V Bock E Lynch MA A novel anti-inflammatory role of NCAM-derived mimetic peptide, FGL Neurobiol. Aging 2010 31 118 128 18468731 10.1016/j.neurobiolaging.2008.03.017

[65] Borcel E Pérez-Alvarez L Herrero AI Brionne T Varea E Berezin V Bock E Sandi C Venero C Chronic stress in adulthood followed by intermittent stress impairs spatial memory and the survival of newborn hippocampal cells in aging animals: Prevention by FGL, a peptide mimetic of neural cell adhesion molecule Behav. Pharmacol 2008 19 41 49 18195593 10.1097/FBP.0b013e3282f3fca9

[66] Popov VI Medvedev NI Kraev IV Gabbott PL Davies HA Lynch M Cowley TR Berezin V Bock E Stewart MG A cell adhesion molecule mimetic, FGL peptide, induces alterations in synapse and dendritic spine structure in the dentate gyrus of aged rats: A three-dimensional ultrastructural study Eur. J. Neurosci 2008 27 301 314 18215229 10.1111/j.1460-9568.2007.06004.x

[67] Aonurm-Helm A Berezin V Bock E Zharkovsky A NCAM-mimetic, FGL peptide, restores disrupted fibroblast growth factor receptor (FGFR) phosphorylation and FGFR mediated signaling in neural cell adhesion molecule (NCAM)-deficient mice Brain Res 2010 1309 1 8 19909731 10.1016/j.brainres.2009.11.003

[68] Anand R Seiberling M Kamtchoua T Pokorny R Tolerability, safety and pharmacokinetics of the FGLL peptide, a novel mimetic of neural cell adhesion molecule, following intranasal administration in healthy volunteers Clin. Pharmacokinet 2007 46 351 358 17375985 10.2165/00003088-200746040-00007

[69] Jacobsen J Kiselyov V Bock E Berezin V A peptide motif from the second fibronectin module of the neural cell adhesion molecule, NCAM, NLIKQDDGGSPIRHY, is a binding site for the FGF receptor Neurochem. Res 2008 33 2532 2539 18368482 10.1007/s11064-008-9680-2

